# Supplementary material for: Characterising the genetic architecture of changes in adiposity during adulthood using electronic health records
Source: Nat Commun. 2024 Jul 10;15:5801. doi: 10.1038/s41467-024-49998-0 (PMC11237142; doi:10.1038/s41467-024-49998-0)
Supplement: Supplementary file 1 — Supplementary Information [file 41467_2024_49998_MOESM1_ESM.pdf]

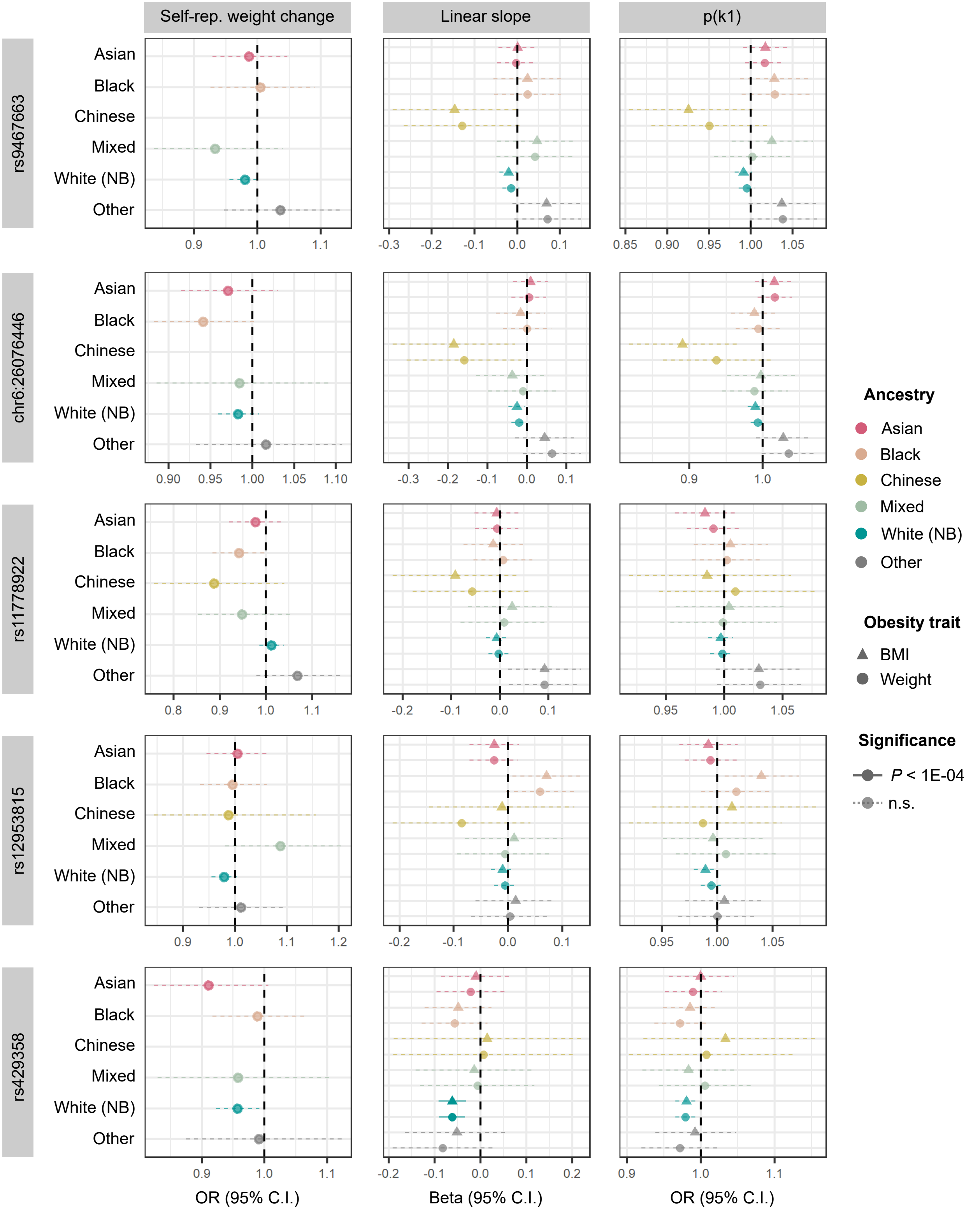

Supp. Figure 1: Association of each of the 5 obesity-change lead SNPs with obesity-change phenotypes in individuals not in the white British ancestry subset of UK Biobank. Left column: Odds ratio (OR) and 95% confidence intervals (C.I.) from proportional odds logistic regression models for association of lead SNP genotypes with self-reported weight change in the past year, coded as an ordinal categorical variable with levels: "Loss", "No change", and "Gain". Middle column: Effect size (beta) and 95% C.I. for associations of lead SNP genotypes with body mass index (BMI) and weight linear slope change over time, estimated from linear mixed effects models. Effect estimates for slope associations are adjusted for intercept value. Right column: Odds ratio (OR) and 95% C.I. for association of lead SNP genotypes with posterior probability of membership in the BMI and weight high gain clusters (k1). All estimates are adjusted for baseline obesity trait value. Points are coloured by self-identified ethnicity (ancestry). White (NB) represents individuals of self-identified white ethnicity not in the genetically determine white British ancestry subset. Solid lines are significant at multiple testing-adjusted P < 1E-04 (Bonferroni corrected for 200 tests), dashed lines are non-significant (n.s.)

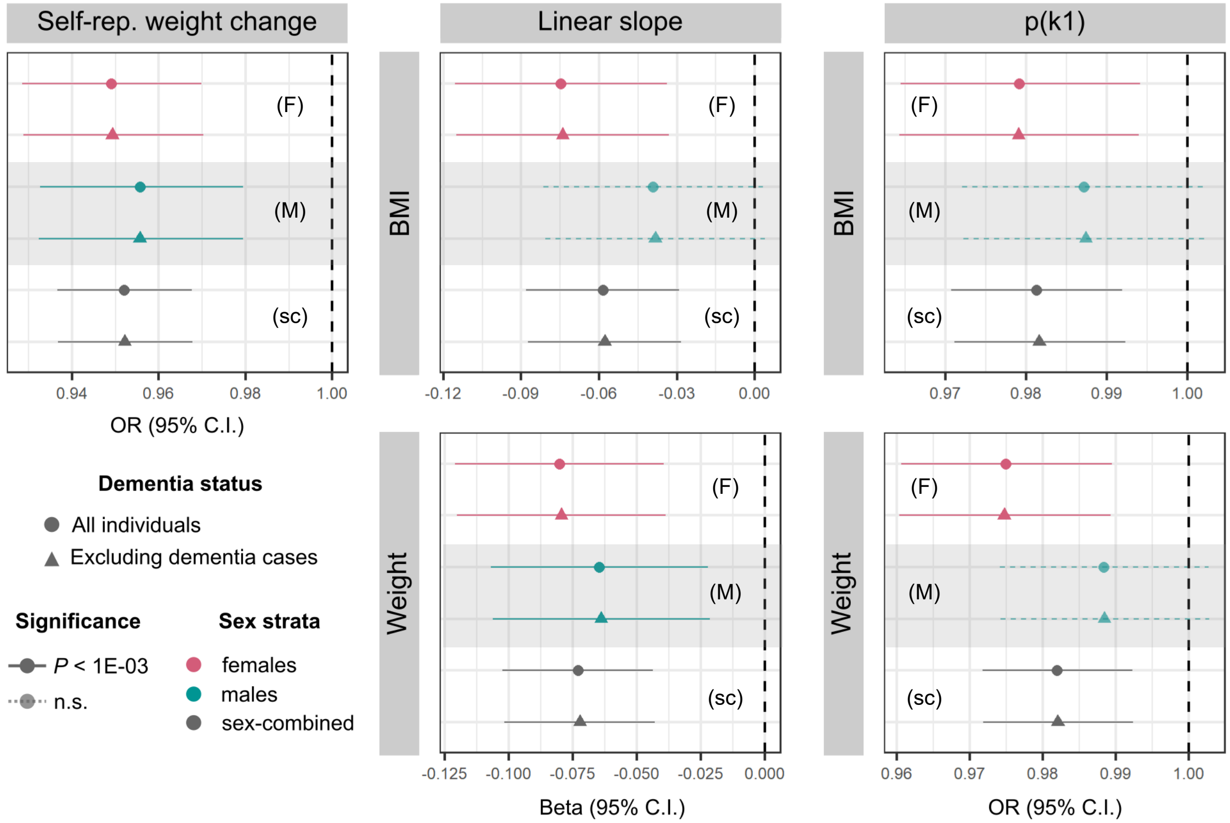

Supp. Figure 2: Association of rs429358 with obesity-change phenotypes in individuals not in the discovery set, and for comparison excluding those with diagnoses of dementia or Alzheimer's. Left column: Odds ratio (OR) and 95% confidence intervals (C.I.) from proportional odds logistic regression models for association of minor C allele of rs429358 with self-reported weight change in the past year, coded as an ordinal categorical variable with levels: "Loss", "No change", and "Gain". Middle column: Effect size (beta) and 95% C.I. for associations of rs429358 genotype with body mass index (BMI) and weight linear slope change over time, estimated from linear mixed effects models. Effect estimates for slope associations are adjusted for intercept value. Right column: Odds ratio (OR) and 95% C.I. for association of rs429358 genotype with posterior probability of membership in the BMI and weight high gain clusters (k1). All estimates are adjusted for baseline obesity trait value. Circles represent associations in all individuals, while triangles are estimated in the subset without dementia or Alzheimer's disease diagnoses. Points are coloured by sex strata. Solid lines are significant at multiple testing-adjusted P < 1E-03 (Bonferroni corrected for 15 tests), dashed lines are non-significant (n.s.).

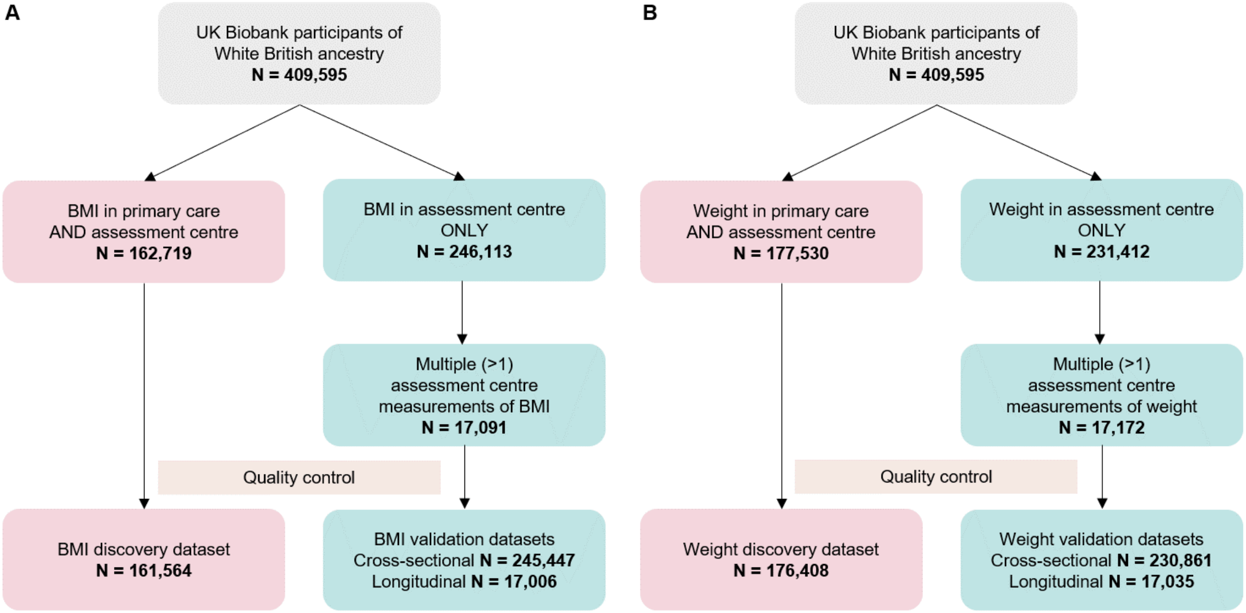

Supp. Figure 3. Selection of discovery and validation datasets from UK Biobank-linked primary care and assessment centre records of (A) body mass index (BMI) and (B) weight.

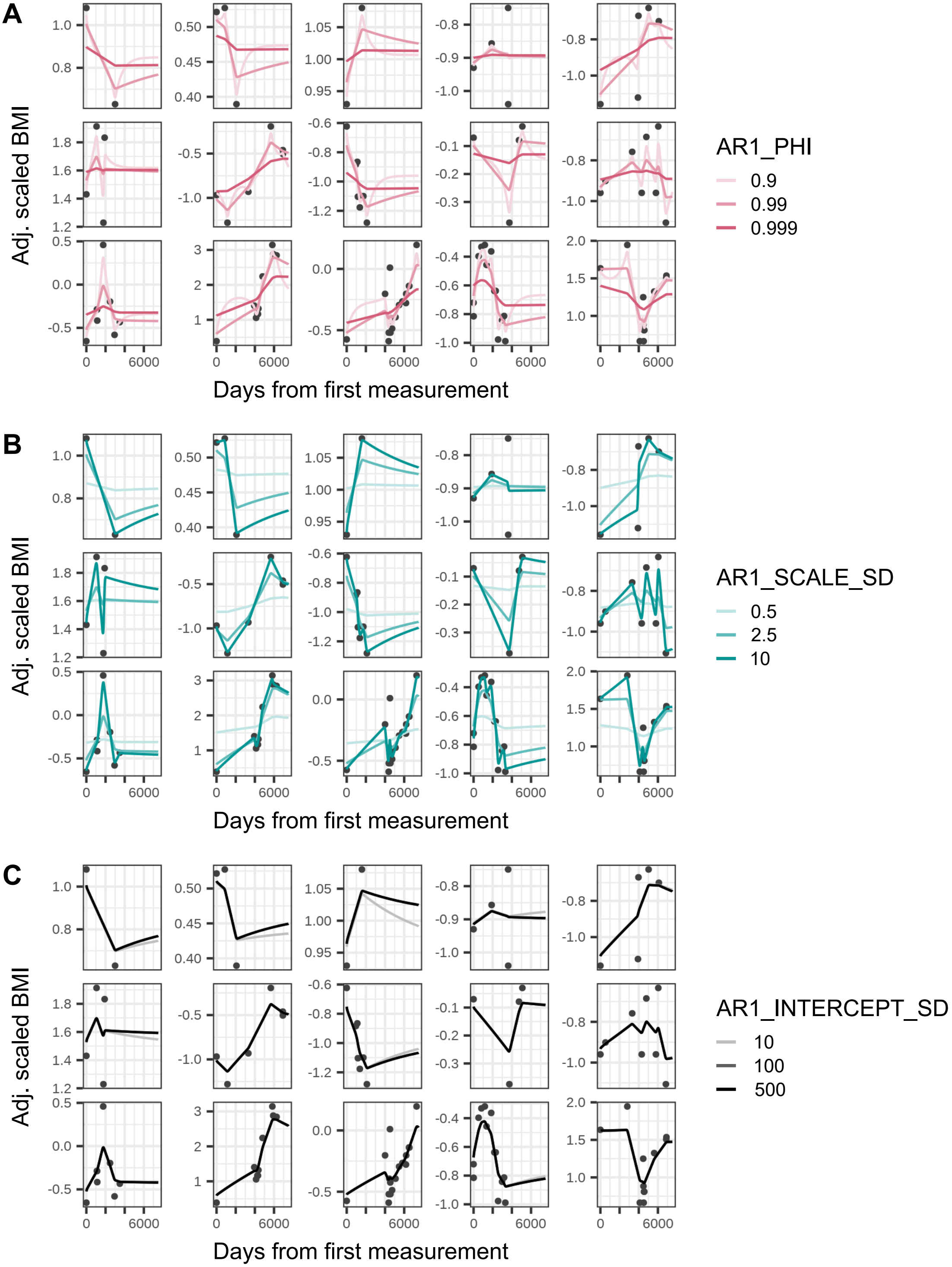

Supp. Figure 4. Selection of hyperparameters for order 1-autoregressive (AR1) model as smoothing prior on spline coefficients. Each facet displays the observed body mass index (BMI) values, adjusted for covariates and normally scaled (see Methods) for a randomly selected individual, in black points. **(A)** AR1 scale standard deviation (SD) fixed to 2.5 and intercept SD to 100, with different coloured lines displaying high-dimensional B-spline model fits at different values of AR1 autocorrelation (phi). **(B)** AR1 autocorrelation fixed to 0.99 and intercept SD to 100, with different coloured lines displaying high-dimensional B-spline model fits at different values of AR1 scale SD. (C) AR1 autocorrelation fixed to 0.99 and scale SD to 2.5, with different coloured lines displaying high-dimensional B-spline model fits at different values of AR1 intercept SD.

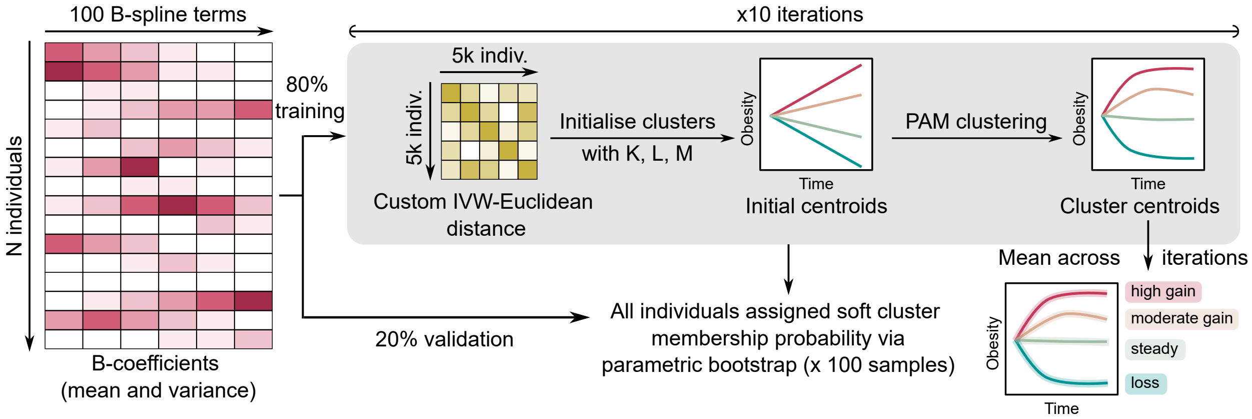

Supp. Figure 5. Overview of clustering protocol. In each analysis strata, individuals are randomly assigned to training (80%) or validation (20%) sets. In each of S = 10 iterations, 5,000 individuals are randomly sub-sampled from the training set for the construction of a custom inverse-variance weighted (IVW) Euclidean distance matrix. Cluster centroids are initialised for each combination of K, L, and M parameters, defined as: K = number of clusters, ranging from 2 to 8, L = filter parameter (filtered to individuals with minimum number of measurements) taking values 2, 5, or 10, and M = initialisation parameter for empirical k-tile of the estimated fold change in obesity trait between baseline and year M with values 1, 2, 5, or 10 years, or random initialisation. Centroids are determined by partitioning around medoids (PAM) clustering and averaged across all 10 iterations after ordering of the non-overlapping and monotonic centroids. All individuals (across training and validation sets) are then assigned soft cluster membership probability for each cluster via a parametric bootstrap across 100 Monte Carlo samples from the B-coefficient posterior.

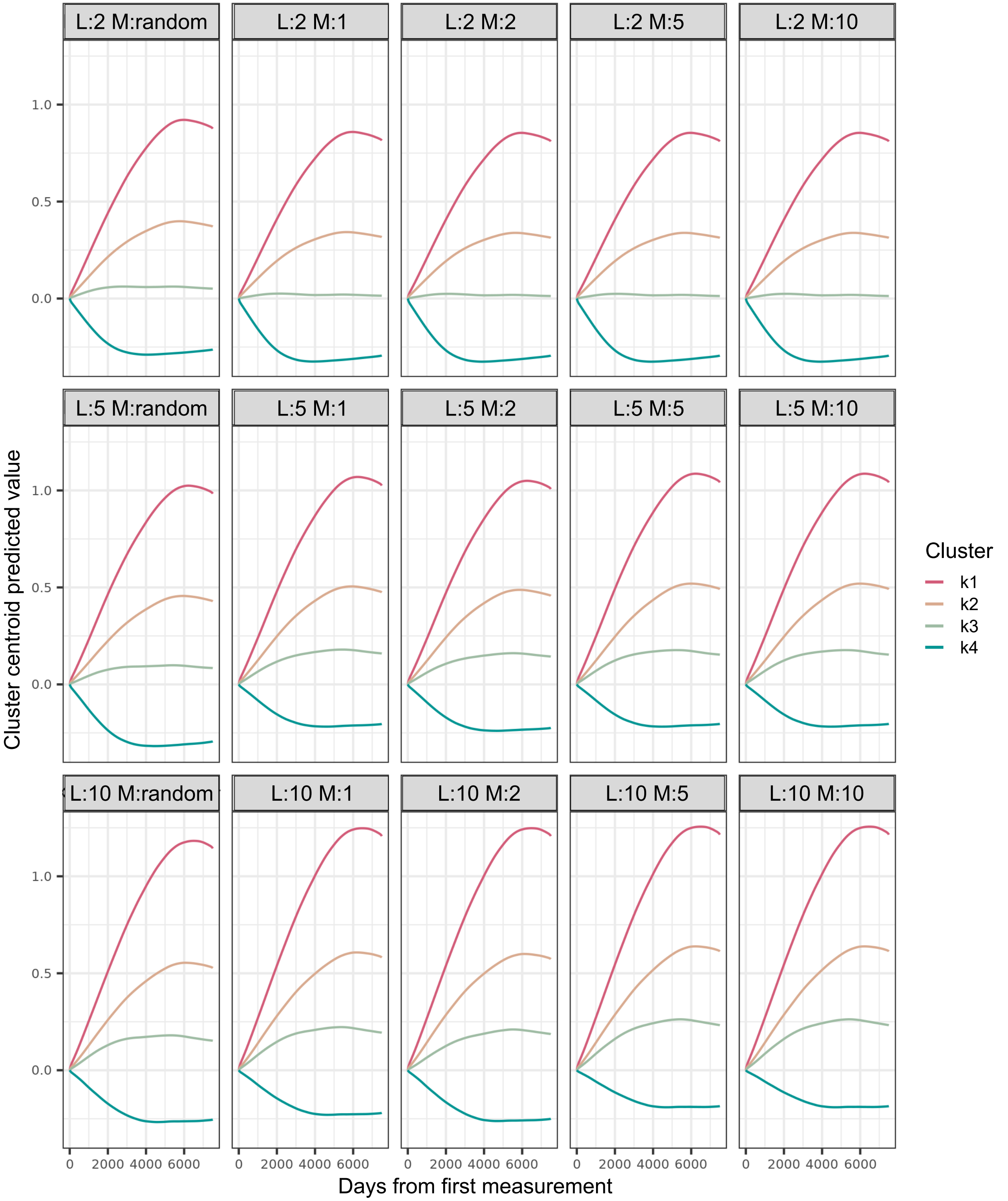

Supp. Figure 6. Cluster centroid trajectories for various combinations of L (filtering) and M (initialisation) parameters. Centroids are averaged over S = 10 iterations as described in the Methods, with clustering parameters K = 4, L = filter parameter (filtered to individuals with minimum number of measurements) taking values 2, 5, or 10, and M = initialisation parameter for empirical k-tile of the estimated fold change in obesity trait between baseline and year M with values 1, 2, 5, or 10 years, or random initialisation. Displayed here are trajectories for cluster centroids calculated from B-spline coefficients from models of body mass index (BMI) in the sex-combined analysis stratum; the y-axis is thus predicted standardised residualised BMI.

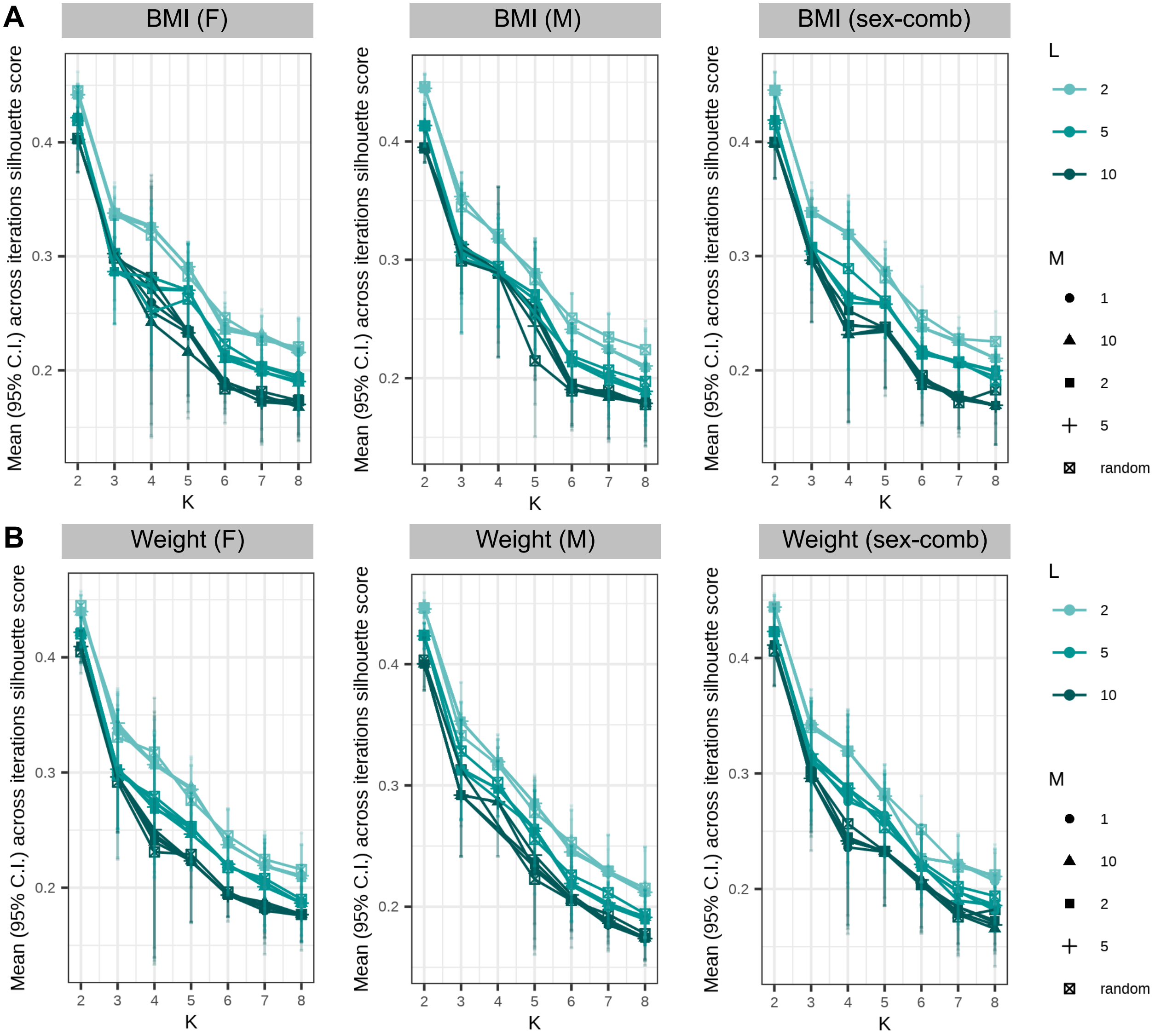

Supp. Figure 7. Silhouette values for selection of K, L, and M clustering parameters. K = number of clusters, ranging from 2 to 8, L = filter parameter (filtered to individuals with minimum number of measurements) taking values 2, 5, or 10, and M = initialisation parameter for empirical k-tile of the estimated fold change in obesity trait between baseline and year M with values 1, 2, 5, or 10 years, or random initialisation. For each combination of parameters, partitioning around medoids (PAM) clustering is run S = 10 times, taking a random sub-sample of 5,000 individuals (subject to filtering parameter) at each iteration. The mean and 95% confidence interval (C.I.) silhouette value across the 10 iterations of clustering with each combination of parameters is plotted. **(A)** Clustering of B-spline coefficients from models of body mass index (BMI) over time in each of the three analysis strata: female (F), males (M), and sex-combined (sex-comb). **(B)** Same as (A), but with weight as the outcome in B-spline models.

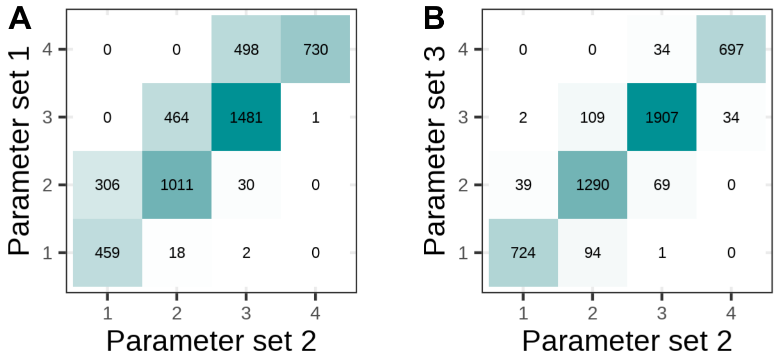

Supp. Figure 8. Comparison of cluster allocations across choice of order 1-autoregressive (AR1) model hyperparameters (BMI sex-combined strata). 5,000 randomly selected individuals were assigned to clusters (see Methods) following high-dimensional B-spline modelling with different values of AR1 model hyperparameters for smoothing of B-spline coefficients. Parameter set 1: AR1 autocorrelation = 0.9, AR1 scale standard deviation (SD) = 0.5, AR1 intercept SD = 10; parameter set 2: AR1 autocorrelation = 0.99, AR1 scale standard deviation (SD) = 2.5, AR1 intercept SD = 100; parameter set 3: AR1 autocorrelation = 0.999, AR1 scale standard deviation (SD) = 10, AR1 intercept SD = 500. The plots display the number of individuals assigned to each of the four clusters (see Methods) following **(A)** modelling with hyperparameter sets 1 and 2; and **(B)** hyperparameter sets 2 and 3 respectively.

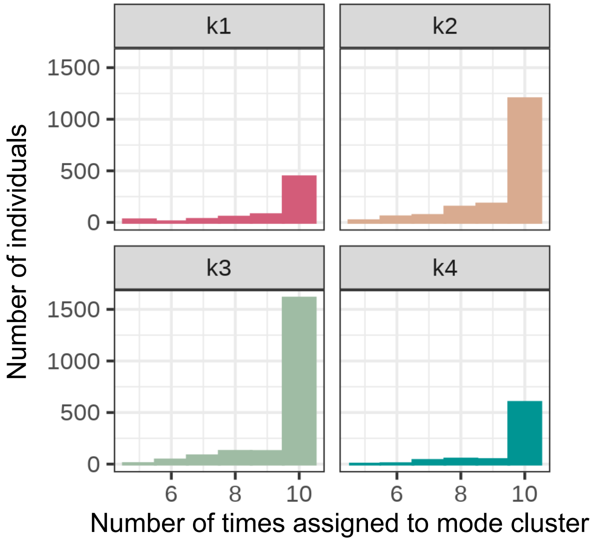

Supp. Figure 9. Comparison of cluster allocations across each of 10 random splits of training data (BMI sex-combined strata). 5,000 randomly selected individuals were held out of clustering training (see Methods) and assigned to the centroids generated in each of 10 random splits of the training data. The histograms display number of times each individual was assigned to their modal cluster - 10 indicates that the individual was assigned to the same cluster in each random split.

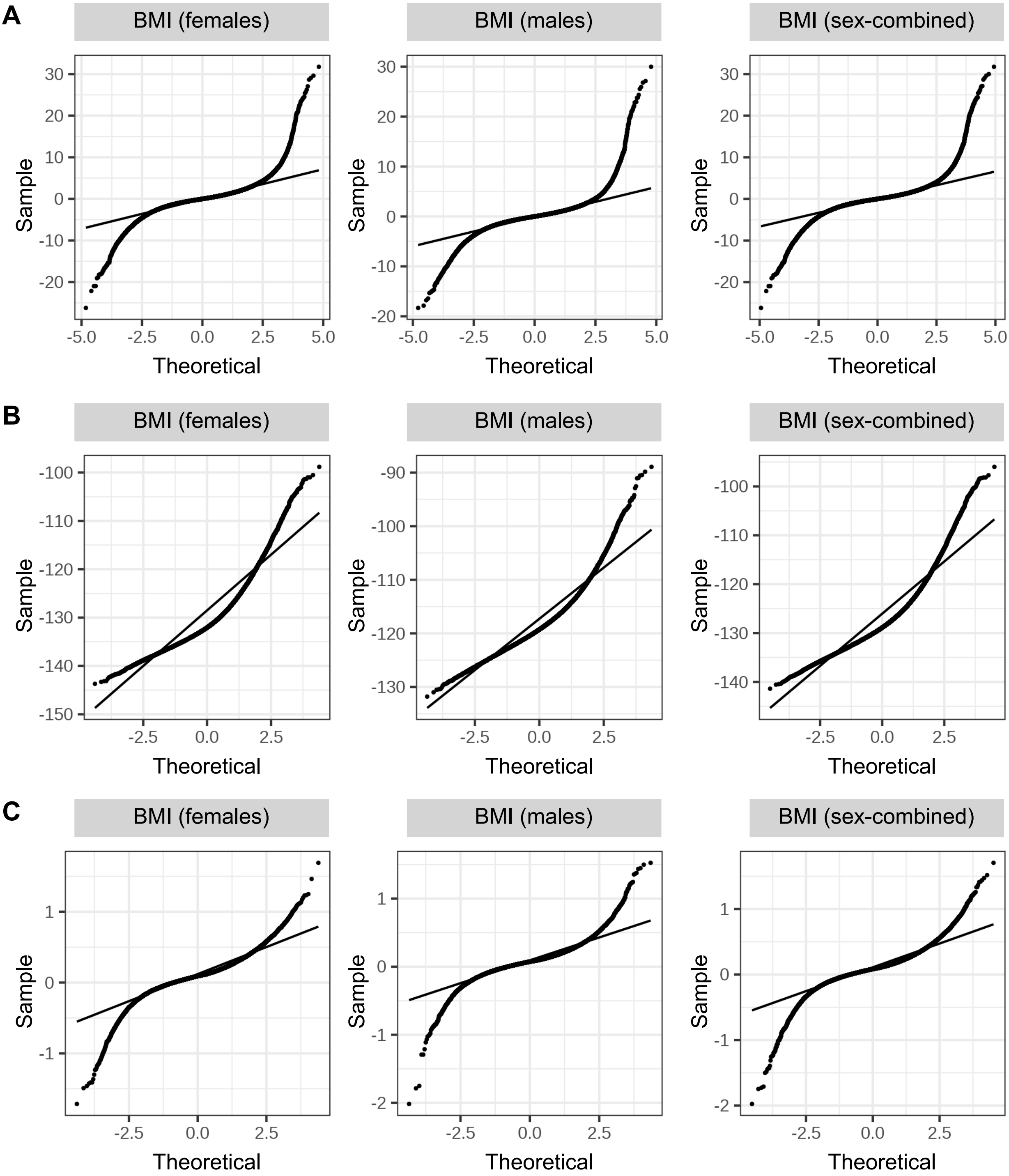

Supp. Figure 10. Quantile-quantile plots for the distribution of residuals and coefficients estimated from linear mixed-effects models of change in body mass index (BMI) over time. (A) Residuals from models in each strata (i.e. females, males, and sex-combined) plotted against theoretical quantiles of the normal distribution. **(B)** QQ-plots for the best linear unbiased predictor (BLUP) estimates of u0, i.e. intercept term, from models in each strata. **(C)** QQ-plots for the BLUP estimates of u1, i.e. slope term, from models in each strata.

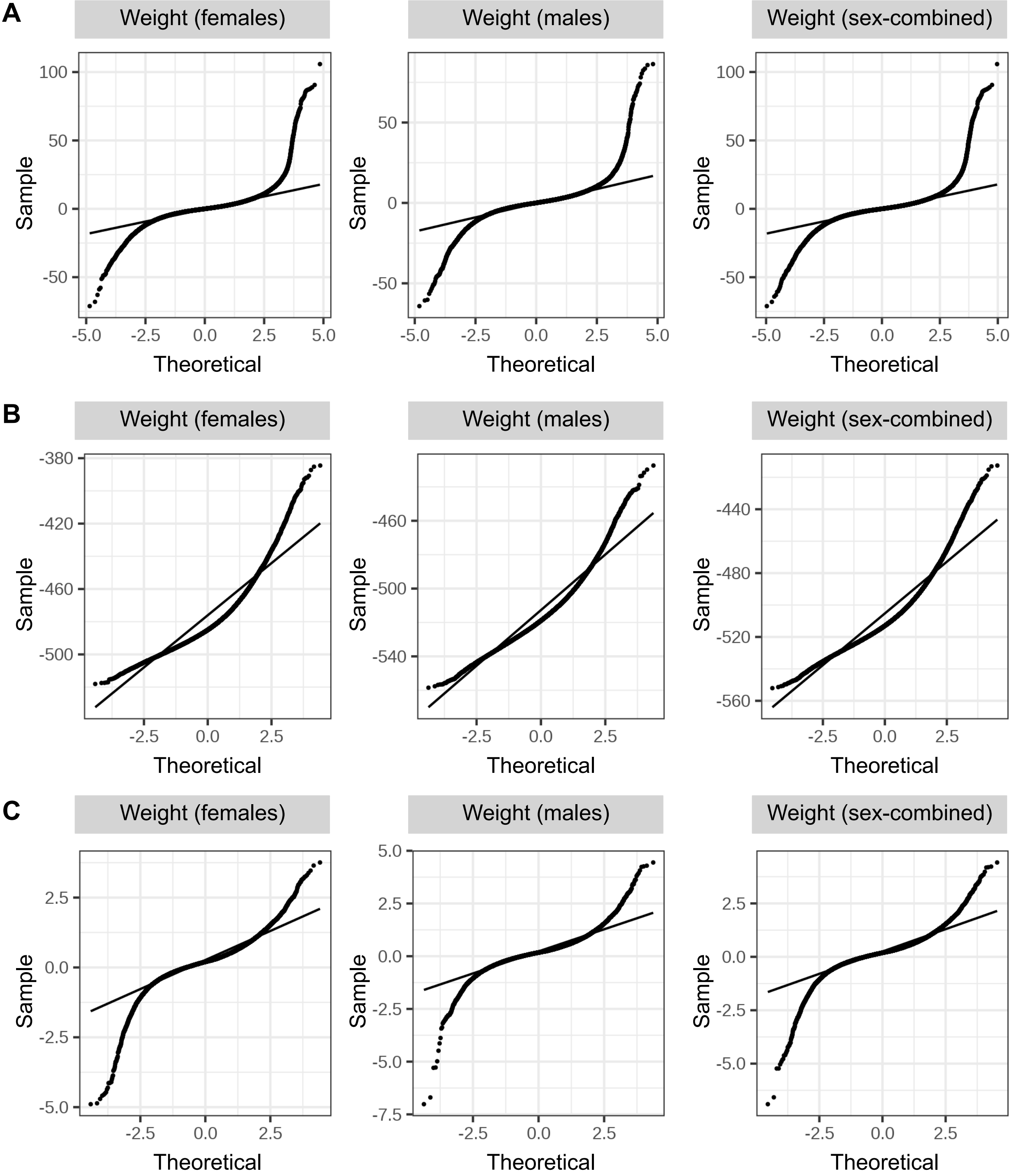

Supp. Figure 11. Quantile-quantile plots for the distribution of residuals and coefficients estimated from linear mixed-effects models of change in weight over time. (A) Residuals from models in each strata (i.e. females, males, and sex-combined) plotted against theoretical quantiles of the normal distribution. **(B)** QQ-plots for the best linear unbiased predictor (BLUP) estimates of u0, i.e. intercept term, from models in each strata. **(C)** QQ-plots for the BLUP estimates of u1, i.e. slope term, from models in each strata.

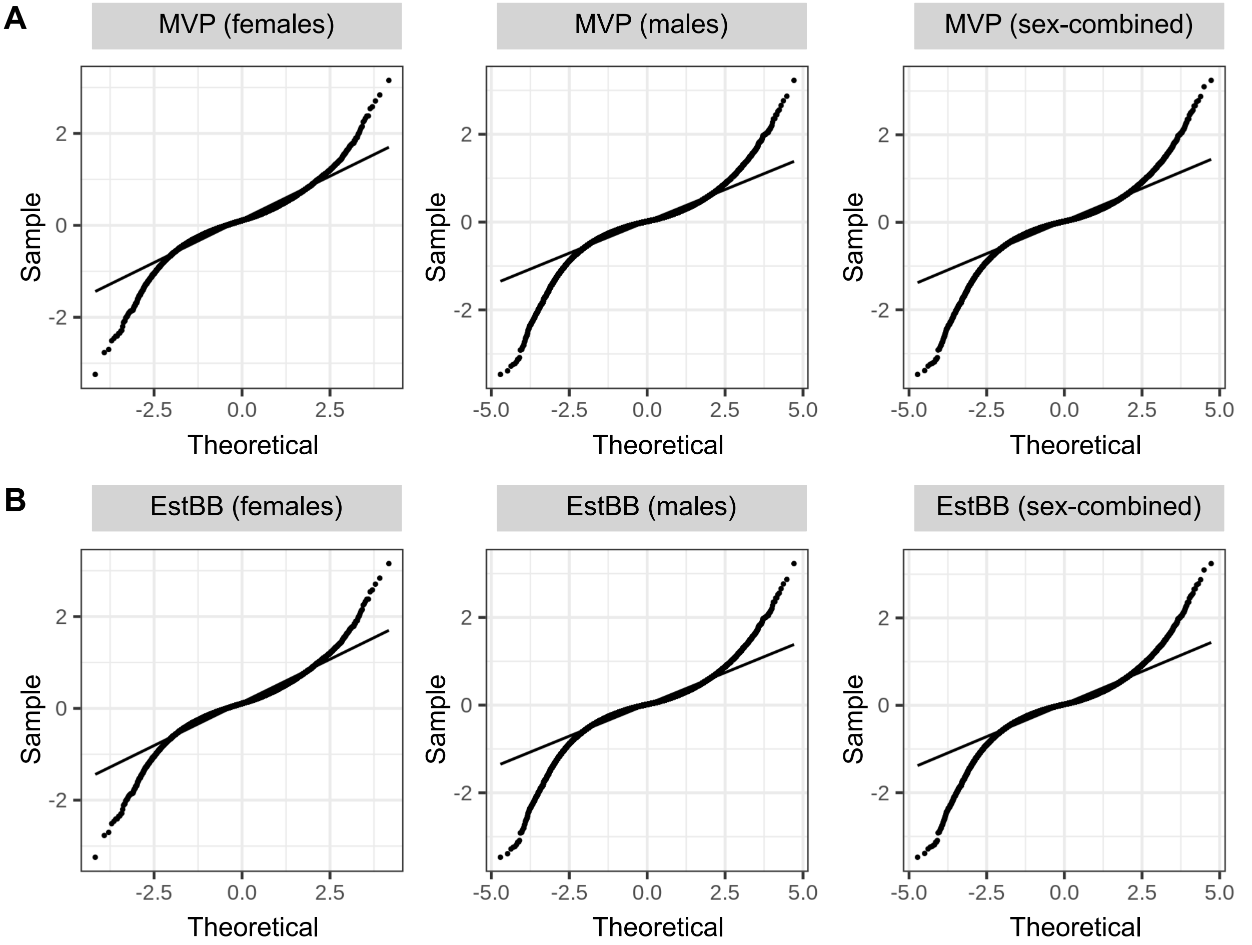

Supp. Figure 12. Quantile-quantile plots for the distribution of best linear unbiased predictor (BLUP) estimates of u1, i.e. slope term, estimated from linear mixed-effects models of change in body mass index (BMI) over time. (A) QQ-plots from the Million Veterans Program (MVP) cohort of up to 437,703 participants. **(B)** QQ-plots from the Estonian Biobank (EstBB) cohort of up to 125,209 participants.

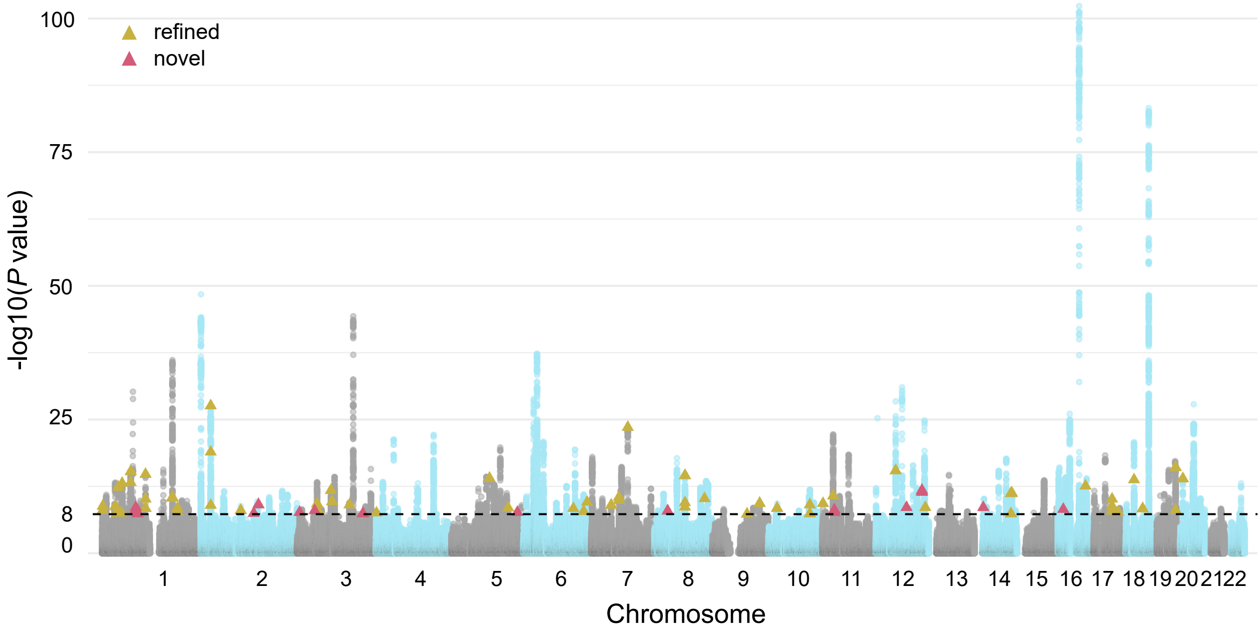

Supp. Figure 13. Genome-wide novel and refined SNP associations with baseline adiposity estimated over the measurement window for each individual. Combined Manhattan plot displaying genome-wide SNP associations with adiposity trait (BMI or weight) across female, male, and sex-combined analysis strata. Each point represents a SNP, with genome-wide significant SNPs (P<5E-8) coloured in yellow for refined SNPs that represent conditionally independent ($P_{conditional}$<0.05) and stronger associations with baseline obesity than published SNPs in the region, and pink for novel associations (see Methods).

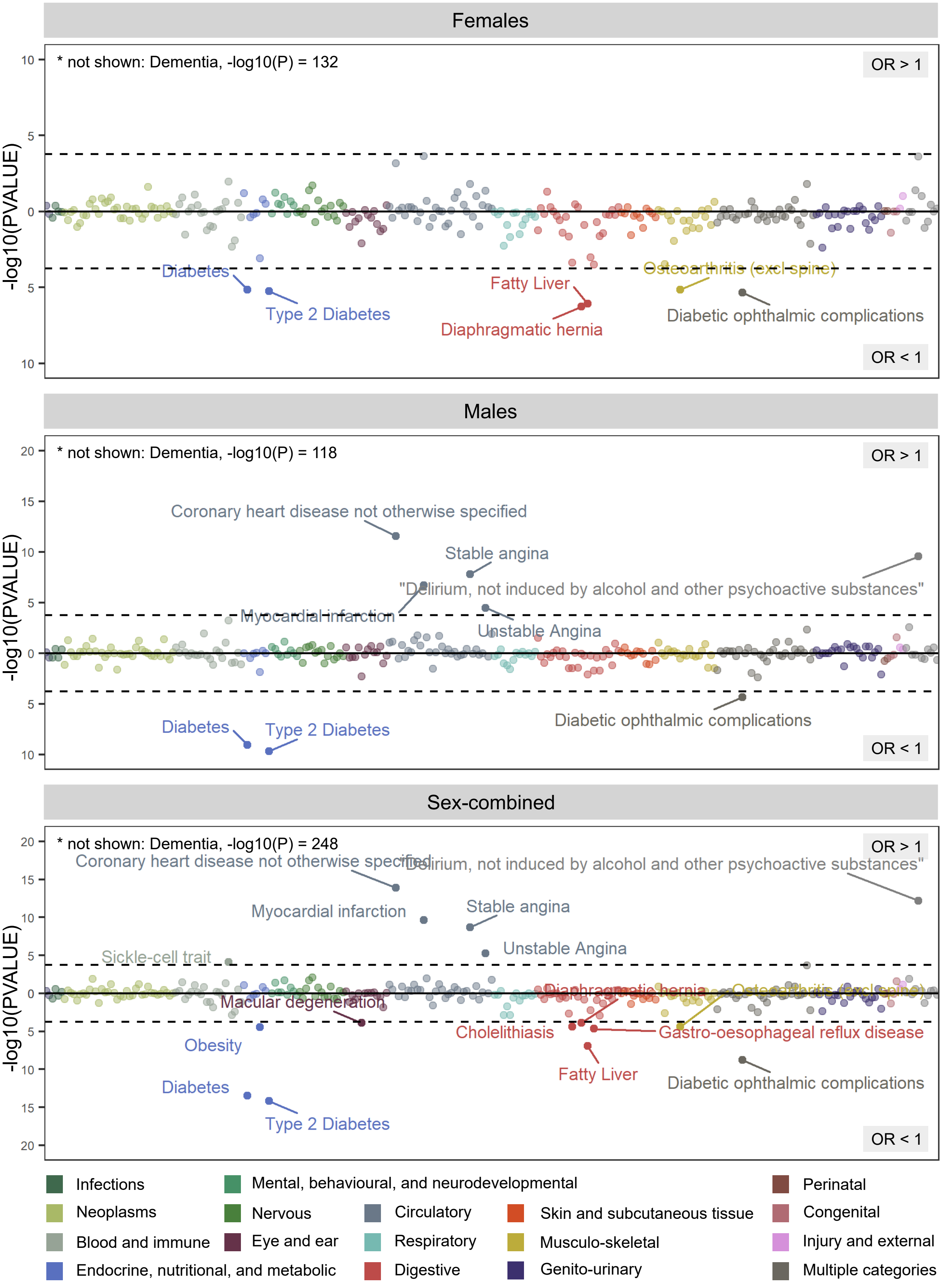

Supp. Figure 14. Phenome-wide associations of rs429358 with diseases in UK Biobank. Logistic regression models were used to assess the association of rs429358 with 290 disease phenotypes, curated from primary care, secondary care, and self-report records in individuals of genetically ascertained White British ancestry the UK Biobank (max sample size ~ 490,000). All associations in the sex-combined analysis were adjusted for sex. The Miami plots display -log10(PVALUE) for significance of the association, with deleterious effects of the minor 'C' allele (odds ratio OR > 1) shown above the horizontal 0 line, and protective effects (OR < 1) shown below the horizontal 0 line. Associations are coloured by ICD chapter, and those significant at P < 0.05/290 are labelled.

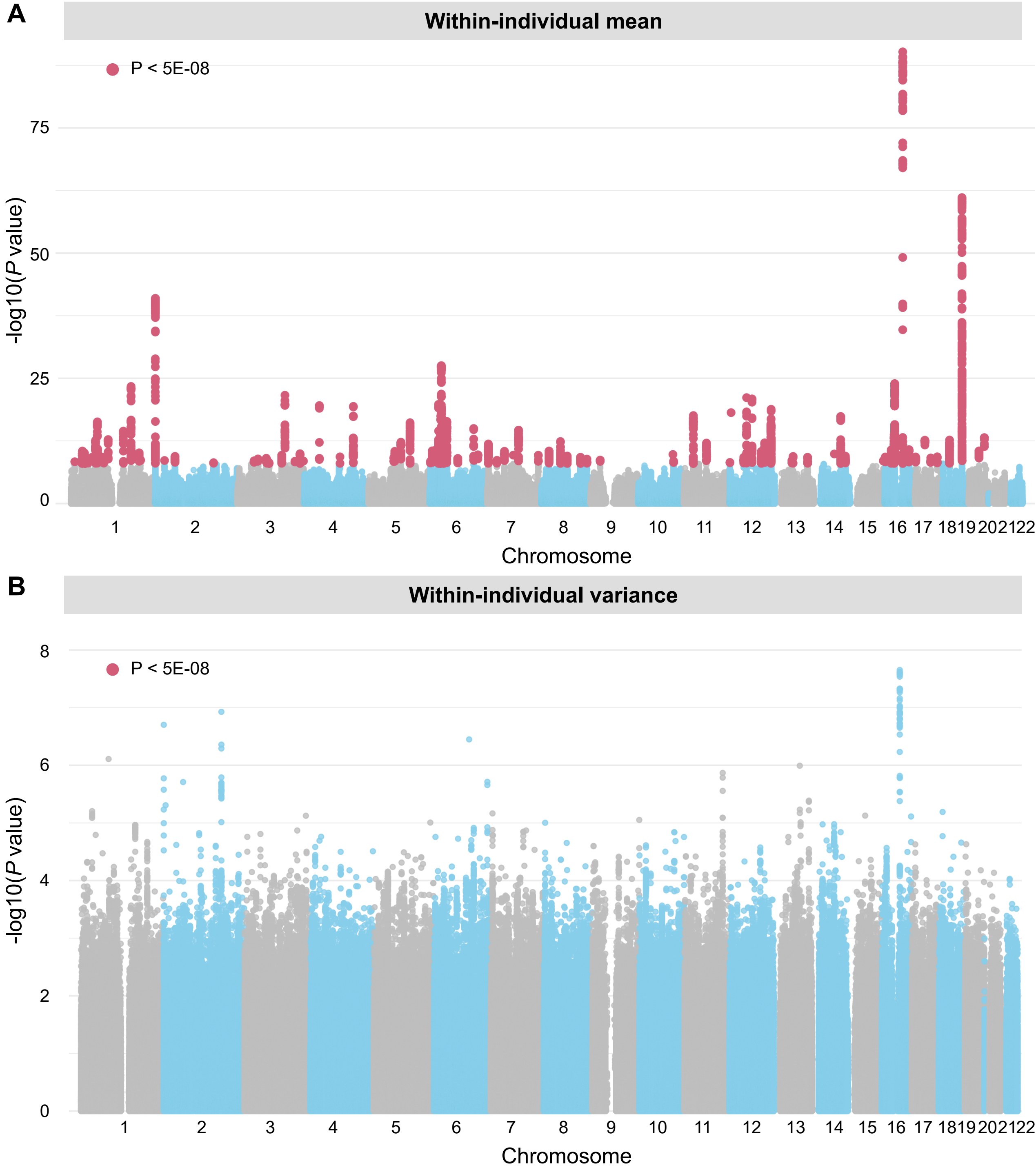

Supp. Figure 15. Manhattan plots for genetic associations with jointly modelled within-individual (A) mean and (B) variance in weight over multiple measurements. Genome-wide association studies (GWASs) for within-individual mean and variance were performed using the TrajGWAS software for 177,472 unrelated male and female participants of white British ancestry with multiple measurements of weight in the UK Biobank (UKBB) and UKBB-linked electronic health records. Each point is a genetic variant, represented along the hg19 chromosome and position x-axis, with -log10(Pvalue) for significance of association with the phenotype on the y-axis. Variants that cross the genome-wide significance threshold of P < 5E-08 are highlighted in pink.

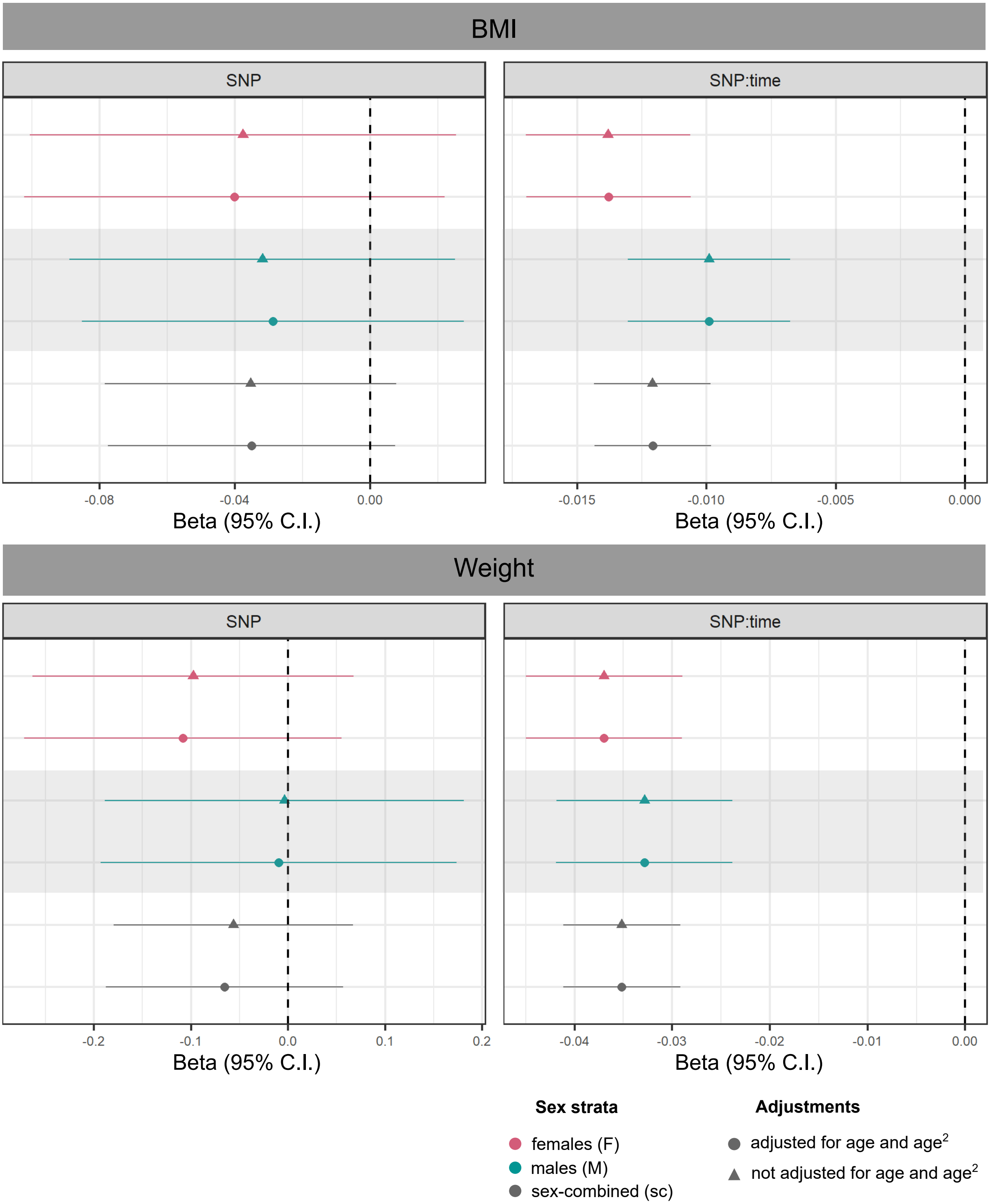

Supp. Figure 16. Comparison of rs429358 effects on baseline adiposity and adiposity-change with and without including baseline age covariates. Displayed are the beta and 95% confidence intervals for the effect of each additional copy of the minor 'C' allele of rs429358 on BMI and weight estimated from linear mixed-effects models in all analysis strata. SNP and SNP interaction with time were included as fixed effects in a linear mixed effects model for adiposity with fixed and random (individual-level) effects of time, with additional adjustments for year of birth, data provider, 21 genetic principal components, and sex (in sex-combined analyses). Effect size estimates for the intercept (SNP) and slope (SNP:time) are displayed for models that were either adjusted for baseline age and age^2 (circles) or not adjusted for these (triangles).

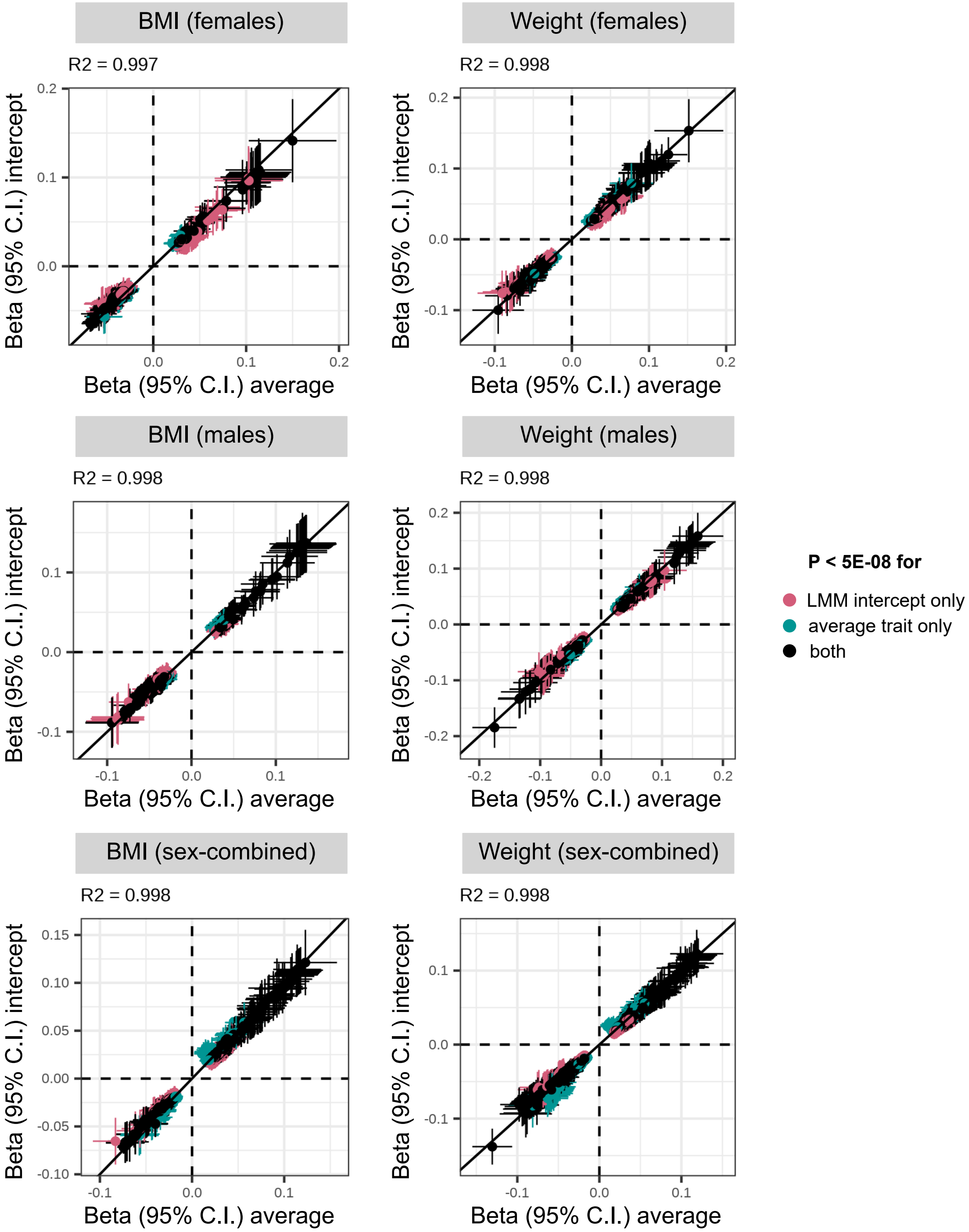

Supp. Figure 17. Comparison of effect sizes of genome-wide significant SNPs in average-adiposity trait and linear mixed model intercept (u0) GWASs. Each facet represents a different analysis strata. SNPs that reach genome-wide significance (P < 5E-08) in GWASs for either u0 (intercept estimated from linear mixed effects models) or average adiposity trait across measurement window for each individual are plotted, coloured by whether SNP is significant in both GWASs or only one. Effect sizes and 95% confidence intervals (CI) are displayed, and correlation (R2) between the two sets of betas is displayed in the top left of each facet.

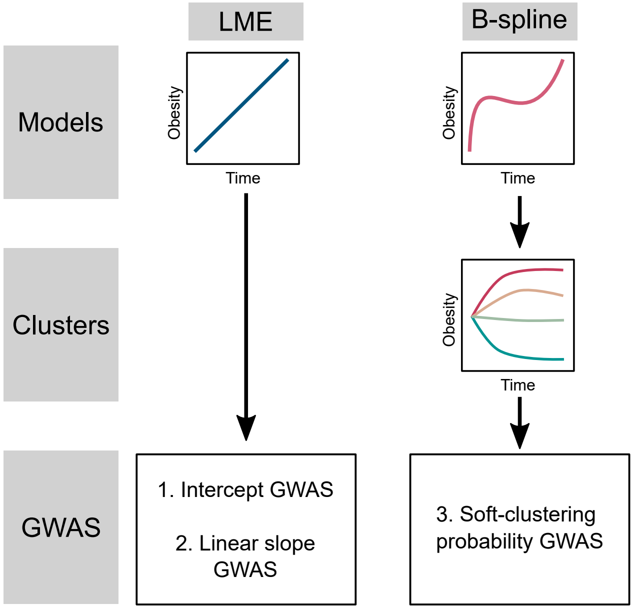

Supp. Figure 18. Workflow for carrying forward estimates from adiposity-change models to GWAS.
