# Supplementary material for: Characterising the genetic architecture of changes in adiposity during adulthood using electronic health records
Source: Nat Commun. 2024 Jul 10;15:5801. doi: 10.1038/s41467-024-49998-0 (PMC11237142; doi:10.1038/s41467-024-49998-0)
Supplement: Supplementary file 3 — Description of Additional Supplementary Files [file 41467_2024_49998_MOESM3_ESM.pdf]

Description of Additional Supplementary Files

File Name: Supplementary Data 1
Description: List of obesity-associated terms in the DISEASE/TRAIT field of the GWAS Catalog [accessed on 02 Nov 2021].

File Name: Supplementary Data 2
Description: Novel and refined SNPs, alongside closest reported obesity-associated SNP, identified from genome-wide association studies (GWAS) of linear mixed effects model intercept. MAF = minor allele frequency (European-ancestry), TSS = transcription start site.

File Name: Supplementary Data 3
Description: Novel genome-wide significant (P<5E-08) BMI and weight SNPs identified in longitudinal intercept GWAS alongside GWAS results from: (1) held-out set of individuals with only UK Biobank assessment centre measurements of obesity, (2) longitudinal intercept GWAS in Million Veterans Program (MVP), (3) longitudinal intercept GWAS in Estonian Biobank, and (4) obesity-related trait look-up from publicly available GWAS summary statistics that include non-UKB participants.

File Name: Supplementary Data 4
Description: Ascertainment (informed presence) bias in primary care (GP) BMI and weight records.

File Name: Supplementary Data 5
Description: Lead genome-wide significant SNPs (P<5E-08) identified from genome-wide association studies (GWAS) of linear slope change of obesity over time, or posterior probability of membership in an obesity cluster (high gain, high/moderate gain, or high/moderate gain and steady), independent of baseline obesity; alongisde replication in held-out set of UK Biobank, Million Veterans Program, and Estonian Biobank. MAF = minor allele frequency (European-ancestry), TSS = transcription start site, S.E. = standard error, OR = odds ratio, C.I. = confidence interval.

File Name: Supplementary Data 6
Description: Effect of rs429358 (minor allele C) on abdominal obesity change, estimated as slope change over time from linear mixed effects models adjusted for intercept, and self-reported 1 year weight change (as ordinal categories, adjusted for BMI), in UK Biobank held-out sets. All effects are estimated with linear regression models.

File Name: Supplementary Data 7
Description: Effect of obesity-change lead SNPs (P<5E-08) on obesity-change phenotypes in individuals not belonging to the white British ancestry subset of UK Biobank. All associations are adjusted for baseline obesity. Blank rows indicate insufficient sample sizes for analysis. Effects are estimated with linear regression models.

File Name: Supplementary Data 8
Description: Association of rs429358 with linear slope change in 45 quantitative biomarker traits, adjusted for baseline trait value. Effects are estimated with linear regression models.

File Name: Supplementary Data 9
Description: Phenotypic and genetic correlation between obesity-change phenotypes.

File Name: Supplementary Data 10
Description: List of read codes to determine date and status of bariatric surgery in primary care records.

File Name: Supplementary Data 11
Description: Characterisation of obesity trait data in longitudinal records curated from UK Biobank assessment centre visits and linked general practitioner (GP) records, non-white British ancestry subset. S.D. = standard deviation, I.Q.R. = inter-quartile range.

File Name: Supplementary Data 12
Description: Median residual variance from high-dimensional spline modelling of standardised obesity trait outcomes, within each strata modelled.

File Name: Supplementary Data 13
Description: Comparison of cluster characteristics in training (80%) vs validation (20%) sets within each analysis strata. Hard cluster assignments were decided as the maximum soft clustering probability of an individual belonging to each cluster. BMI = body mass index, S.D. = standard deviation, I.Q.R. = interquartile range.

File Name: Supplementary Data 14
Description: List of codes to determine quantitative trait (anthropometric traits and biomarkers) values from UK Biobank assessment centres (field codes) and GP-linked data (read codes).

File Name: Supplementary Data 15
Description: List of read codes and ICD codes to determine history or diagnosis of dementia or Alzheimer's disease in primary care and secondary care records.

File Name: Supplementary Data 16
Description: Look-ups of longevity-associated SNPs from GWAS Catalog in adiposity-change GWAS summary statistics.

File Name: Supplementary Data 17
Description: Association of adiposity-change lead SNPs (P<5E-08) with linear slope change in 45 quantitative biomarker traits, adjusted for baseline trait value. All effects are estimated with linear regression models.

File Name: Supplementary Data 18
Description: Effect of adiposity-change lead SNPs (P<5E-08) on abdominal adiposity change, estimated as slope change over time from linear mixed effects models adjusted for intercept, and self-reported 1 year weight change (adjusted for BMI), in UK Biobank held-out sets. All effects are estimated with linear regression models.

File Name: Supplementary Data 19
Description: Lead SNPs (P<5E-08) identified from genome-wide association studies (GWAS) of linear slope change of obesity over time, or posterior probability of membership in an obesity cluster (high gain, high/moderate gain, or high/moderate gain and steady), independent of baseline obesity; alongside replication in held-out set of UK Biobank. MAF = minor allele frequency (European-ancestry), TSS = transcription start site, S.E. = standard error, OR = odds ratio, C.I. = confidence interval.

File Name: Supplementary Data 20
Description: Comparison between linear mixed model intercepts (u0) and average adiposity trait across measurement window.

File Name: Supplementary Data 21
Description: Comparison of lead SNPs identified from genome-wide association studies (GWAS) of linear slope change of obesity over time, or posterior probability of membership in an obesity cluster (high gain, high/moderate gain, or high/moderate gain and steady), independent of baseline obesity. GWASs being compared are either adjusted for number of follow-up measures and length of follow-up, or not adjusted for these metrics. MAF = minor allele frequency, TSS = transcription start site, S.E. = standard error, OR = odds ratio, C.I. = confidence interval.

File Name: Supplementary Data 22
Description: Association between rs429358 allele dosage and follow-up metrics in UK Biobank. Estimates displayed per additional copy of the minor 'C' allele, estimated with linear regression models.

File Name: Supplementary Data 23
Description: Characterisation of BMI in longitudinal records curated from (1) UK Biobank assessment centre visits and linked general practitioner (GP) records, (2) Million Veterans Program cohort, and (3) Estonian Biobank records. S.D. = standard deviation, I.Q.R. = inter-quartile range.

File Name: Supplementary Data 24
Description: Comparison of cluster characteristics in UK Biobank (UKBB), Million Veterans Program (MVP), and Estonian Biobank (EstBB) within each analysis strata. Hard cluster assignments were decided as the maximum soft clustering probability of an individual belonging to each cluster. BMI = body mass index, S.D. = standard deviation, I.Q.R. = interquartile range.

File Name: Supplementary Data 25
Description: sizes. Replication alpha set to 0.05/6 (Bonferroni-adjusted for 6 independent variants). MAF = minor allele frequency, SE = standard error.

File Name: Supplementary Data 26
Description: GWAS Catalog Accession numbers to download summary statistics. For example, to download summary statistics for trait BMI_F_b0, use: ftp://ftp.ebi.ac.uk/pub/databases/gwas/summary_statistics/GCST90429001-GCST90430000/GCST90429765
