# Supplementary material for: Characterising the genetic architecture of changes in adiposity during adulthood using electronic health records
Source: Nat Commun. 2024 Jul 10;15:5801. doi: 10.1038/s41467-024-49998-0 (PMC11237142; doi:10.1038/s41467-024-49998-0)
Supplement: Supplementary file 5 — Reporting Summary [file 41467_2024_49998_MOESM5_ESM.pdf]

Corresponding author(s): Samvida S. Venkatesh, Cecilia M. Lindgren, George Nicholson

Last updated by author(s): 30/11/23

Reporting Summary

Nature Portfolio wishes to improve the reproducibility of the work that we publish. This form provides structure for consistency and transparency in reporting. For further information on Nature Portfolio policies, see our Editorial Policies and the Editorial Policy Checklist.

Statistics

For all statistical analyses, confirm that the following items are present in the figure legend, table legend, main text, or Methods section.

n/a	Confirmed	
☐	☒	The exact sample size (*n*) for each experimental group/condition, given as a discrete number and unit of measurement
☐	☒	A statement on whether measurements were taken from distinct samples or whether the same sample was measured repeatedly
☐	☒	The statistical test(s) used AND whether they are one- or two-sided<br>*Only common tests should be described solely by name; describe more complex techniques in the Methods section.*
☐	☒	A description of all covariates tested
☐	☒	A description of any assumptions or corrections, such as tests of normality and adjustment for multiple comparisons
☐	☒	A full description of the statistical parameters including central tendency (e.g. means) or other basic estimates (e.g. regression coefficient) AND variation (e.g. standard deviation) or associated estimates of uncertainty (e.g. confidence intervals)
☐	☒	For null hypothesis testing, the test statistic (e.g. *F*, *t*, *r*) with confidence intervals, effect sizes, degrees of freedom and *P* value noted<br>*Give P values as exact values whenever suitable.*
☐	☒	For Bayesian analysis, information on the choice of priors and Markov chain Monte Carlo settings
☐	☒	For hierarchical and complex designs, identification of the appropriate level for tests and full reporting of outcomes
☐	☒	Estimates of effect sizes (e.g. Cohen's *d*, Pearson's *r*), indicating how they were calculated

Our web collection on statistics for biologists contains articles on many of the points above.

Software and code

Policy information about availability of computer code

Data collection	N/A
Data analysis	The discovery analyses for this study were primarily conducted in R, and genetic association studies were performed in BOLT-LMM, both of which are freely available and commonly used software. All code for analyses are available on GitHub, the link to which is provided in the manuscript; while the repository is currently private, it will be published with the manuscript.<br>Replication genetic association studies in the Million Veteran Program and Estonian Biobank were performed in REGENIE, which is a freely available and commonly used software.

For manuscripts utilizing custom algorithms or software that are central to the research but not yet described in published literature, software must be made available to editors and reviewers. We strongly encourage code deposition in a community repository (e.g. GitHub). See the Nature Portfolio guidelines for submitting code & software for further information.

Data

Policy information about availability of data

All manuscripts must include a data availability statement. This statement should provide the following information, where applicable:

- Accession codes, unique identifiers, or web links for publicly available datasets
- A description of any restrictions on data availability
- For clinical datasets or third party data, please ensure that the statement adheres to our policy

The discovery study is based on UK Biobank data, which is available to approved researchers. Discovery GWAS summary statistics generated in the study will be made publicly available through the GWAS Catalog upon publication.
Replication analyses were conducted in: (1) the Million Veteran Program, which is currently available to Veterans Affair (VA) investigators and other approved partners; and (2) the Estonian Biobank, which can be accessed for scientific research by application to the Scientific Advisory Committee of the Estonian Biobank.

Human research participants

Policy information about studies involving human research participants and Sex and Gender in Research.

Reporting on sex and gender	We use the term "sex" throughout the paper to refer to inferred genetic sex (in the UK Biobank, as inferred by Bycroft et al. 2018, from relative intensity of markers on the X and Y chromosomes), having performed quality control to exclude individuals with sex chromosome aneuploidies and those whose self-reported sex does not match inferred genetic sex. All of our discovery and replication analyses are performed in sex-specific (men only, or women only) and sex-combined strata adjusted for sex. The number of men and women included in each analysis is reported in the manuscript tables; the main analyses have up to 97,000 women and 81,000 men.
Population characteristics	The discovery study is based on the UK Biobank cohort, whose demographic and genotypic characteristics have been detailed in Bycroft et al. 2018. The main analyses were performed in individuals of the White British ancestry subset (as identified via genetic principal components by Bycroft et al. 2018), and supplementary analyses were performed in individuals of other ancestral groups (self-reported).<br>The replication studies were performed in: (1) the Million Veteran Program, whose cohort characteristics have been described in detail by Gaziano et al. 2016; replication was performed in the genetically ascertained European ancestry subset of the cohort, which is 90% male. (2) the Estonian Biobank, whose cohort characteristics have been described in detail by Leitsalu et al. 2015.
Recruitment	The discovery study is based on the UK Biobank cohort with a "healthy volunteer" selection bias, as the cohort is ascertained for women, and older and healthier participants who live in less socio-economically deprived areas than the population of the UK (Fry et al. 2017). This limitation is acknowledged in the manuscript.<br>Replication analyses are performed in the: 1) the Million Veteran Program, which recruits from the 6.9 million eligible individuals who make use of the services provided by the Veterans Health Administration from around 50 Veterans Affair facilities across the USA (Gaziano et al. 2016); 90% of participants are male, and are at higher risk of cardiovascular disease and more overweight and obese than the population of the USA. (2) the Estonian Biobank, which is a volunteer-based sample of Estonian residents comprising about 20% of the Estonian adult population, recruited by medical personnel and through media campaigns. The cohort is representative of the demographic characteristics of the general population of Estonia.
Ethics oversight	UK Biobank has approval from the North West Multi-centre Research Ethics Committee (MREC) as a Research Tissue Bank (RTB) approval.<br>Consent for the Million Veteran Program is obtained in accordance with all Veterans Affairs policies and under the authority of the Veterans Affairs Central IRB.<br>The activities of the Estonian Biobank are regulated by the Human Genes Research Act, which was adopted in 2000 specifically for the operations of the EstBB. Individual level data analysis in the EstBB was carried out under ethical approvals of 1.1-12/1409 and 1.1-12/2161 from the Estonian Committee on Bioethics and Human Research (Estonian Ministry of Social Affairs), using data according to release application 6-7/GI/31993 from the Estonian Biobank.

Note that full information on the approval of the study protocol must also be provided in the manuscript.

Field-specific reporting

Please select the one below that is the best fit for your research. If you are not sure, read the appropriate sections before making your selection.

☒ Life sciences ☐ Behavioural & social sciences ☐ Ecological, evolutionary & environmental sciences

For a reference copy of the document with all sections, see nature.com/documents/nr-reporting-summary-flat.pdf

Life sciences study design

All studies must disclose on these points even when the disclosure is negative.

Sample size	Sample sizes for each analysis are detailed in the manuscript, and the maximum sample size available in each cohort was selected for the study.
Data exclusions	We only excluded data for quality control reasons, i.e. individuals who did not pass standard genotyping quality control measures (based on sex mismatches, relatedness, heterozygosity, etc.) which are detailed in the manuscript. We also performed quality control on the repeat body mass index (BMI) and weight measurements extracted from primary care electronic health records at both the population-level and individual-levels (details in manuscript) to exclude any likely measurement errors.
Replication	We replicated genetic findings in: (1) the held-out subset of the UK Biobank (between 20,000 to 200,000 individuals depending on the analysis) without primary care electronic health records who were not included in the main analyses. We also replicated the clustering of individuals into different adiposity-change pattern groupings in the ~20,000 individuals without primary care records for whom multiple measurements of weight or BMI were available through the UK Biobank assessment centres. (2) The Million Veteran Program cohort, and (3) the Estonian Biobank.<br><br>Details of all replication are provided in the manuscript.
Randomization	N/A - no analyses required randomization.
Blinding	N/A - no analyses required blinding.

Reporting for specific materials, systems and methods

We require information from authors about some types of materials, experimental systems and methods used in many studies. Here, indicate whether each material, system or method listed is relevant to your study. If you are not sure if a list item applies to your research, read the appropriate section before selecting a response.

Materials & experimental systems

n/a	Involved in the study	
☒	☐	Antibodies
☒	☐	Eukaryotic cell lines
☒	☐	Palaeontology and archaeology
☒	☐	Animals and other organisms
☒	☐	Clinical data
☒	☐	Dual use research of concern

Methods

n/a	Involved in the study	
☒	☐	ChIP-seq
☒	☐	Flow cytometry
☒	☐	MRI-based neuroimaging
